# A Novel Type Room Temperature Surface Photovoltage Gas Sensor Device

**DOI:** 10.3390/s18092919

**Published:** 2018-09-03

**Authors:** Monika Kwoka, Michal A. Borysiewicz, Pawel Tomkiewicz, Anna Piotrowska, Jacek Szuber

**Affiliations:** 1 Institute of Electronics, Silesian University of Technology, 44-100 Gliwice, Poland; jacek.szuber@polsl.pl; 2 Institute of Electron Technology, 02-668 Warsaw, Poland; mbory@ite.waw.pl (M.A.B.); ania@ite.waw.pl (A.P.); 3 SemiInstruments Company, 41-800 Zabrze, Poland; ptomkiewicz@semiinstruments.com

**Keywords:** room temperature gas sensor, surface photovoltage effect, porous ZnO nanostructured thin films

## Abstract

In this paper a novel type of a highly sensitive gas sensor device based on the surface photovoltage effect is described. It is based on the Kelvin probe approach. Porous ZnO nanostructured thin films deposited by the direct current (DC) reactive magnetron sputtering method are used as the active gas sensing electrode material. Crucially, the obtained gas sensing material exhibited a nanocoral surface morphology and surface Zn to O non-stoichiometry with respect to its bulk mass. Among other responses, the demonstrated SPV gas sensor device exhibits a high response to an NO_2_ concentration as low as 1 ppm, with a signal to noise ratio of about 50 and a fast response time of several seconds under room temperature conditions.

## 1. Introduction

Despite more than 50 years of development, the most common resistive type gas sensors systems based on metal oxide (MOX) materials still exhibit some critical and fundamental limitations [1,2,3,4], which can be divided into two groups: the first one concerns the analytically useful characteristics which are limited to good sensitivity (usually at the level of single ppm, strongly depending on the gas) combined with a rather poor selectivity (strongly dependent on humidity, that can be improved by noble catalytic metals), as well as poor dynamic parameters such as a long response time (tens of seconds) combined with a rather very long recovery time (single minutes). The latter is especially relevant when compared to the responsiveness of animal and human olfaction [5,6].

The second group concerns mainly the high temperature working conditions, usually in the range of 200 ÷ 400 °C causing high power consumption of these gas sensor devices, combined with their limited reversibility (stability), adversely affecting their costs of fabrication and possible commercialization [7]. However, lately it was observed that low sensing effect of resistive type MOX gas sensors at room temperature can be improved by additional UV radiation, as nicely reviewed, mainly for various forms of ZnO gas sensors, among others, by Zhu and Zeng [8] and by Leonardi [9]. Nevertheless, the sensor requires an additional external source of continuous significant energy (thermal, UV) for stable operation.

At this moment it should be underlined that in the last several years some innovative ideas have appeared in the literature about how to improve the sensing abilities of the resistive type MOX gas sensors related to their abovementioned basic limitations, including some still unsolved technical and technological problems.

The first way concerns the application of various dimensionalities of the gas sensing materials. Nanoscale MOX materials and fabrication technologies have allowed the fabrication of novel type sensing architectures, what additionally helped to broaden the fundamental understanding of their sensing mechanism [10,11,12,13]. However, despite more than one decade of studies the useful MOX characteristics including sensitivity (only about one order better with respect to 3D thin films or even 2D nanolayers) and selectivity combined with the dynamic parameters such as response/recovery times (only slightly shorter with respect to 3D thin films/2D nanolayers) are still below the common expectations [12,13].

The second way concerns the application of selected metal oxides for specific gases based on their better specific response in terms of the abovementioned analytical characteristics. It should be underlined that up to now tin dioxide (SnO_2_) is the most popular gas sensor material (~40% of papers, 60% of commercial gas sensors). However, in the last few years zinc oxide (ZnO) has appeared as second common gas sensor material (~20% papers). This is due to the fact that its electron mobility is at the level of ~10^2^ cm^2^/V·s similar to SnO_2_, while the electron conductivity is at the level of 10^3^ 1/Ω·cm, what is one order higher than SnO_2_ [12,13,14,15].

The third way concerns the use of novel type sensing transduction principles or innovative transducers using flexible sensors with smart textiles, as well as new sensing architectures including Schottky-contact nanosensors, FET chemical sensors, surface ionization sensors and magnetic gas sensors, as recently reviewed by Comini [16].

However, within these ideas the influence of the work function variation and related effects on gas sensing have been ignored. Perhaps this is related to the fact that these effects are based essentially on the variation of contact potential difference (CPD) using mainly Kelvin vibrating capacitor, what was already commonly used as the gas sensors transduction for many years [17,18]. Moreover, it is also well known that this method exhibits a rather poor sensitivity because of a low signal to noise (S/N) ratio, and that this method was mainly applied for studies of porous semiconductor materials [19,20,21]. This is probably the reason why up to now only an incomprehensibly weak attention was given to the use in gas sensors of the effect of work function variation of gas sensing materials (including MOX) after external optical excitation in the so called surface photovoltage effect [22].

In general, the surface photovoltage (SPV) effect appears as a result of variation of the surface potential barrier at the semiconductor surface upon illumination, what is directly related to a photon induced charge generation combined with its redistribution within the space charge region (SCR) at the semiconductor surface. During the gas adsorption/desorption like NO_2_ used in our experiments, which exhibits a dipole nature, the specific and very strong charging/discharging effects at the surface semiconductor are observed, what directly causes the above mentioned variation of the surface potential barrier. It mainly depends on the incident photon energy with respect to the band-gap of semiconductor gas sensor material under investigation and the light intensity [22].

Even sub-band gap photons can modify the charge at the semiconductor surfaces by exciting the trapped carriers at a well defined energy, i.e., when using the photons of energy *hν* = *E_C_* − *E_A_* or *hν* = *E_A_* − *E_V_*, where *E_C_* and *E_V_* are the energy of bottom of conductive band and the top of valence band at the surface, respectively, whereas the *E_A_* is the energy level of traps induced by adsorbed gas species, the specific electron transitions from a surface state to the conduction band (or from the valence band to the surface states) can be observed. In both cases the variation of band bending at the semiconductor surfaces is observed, corresponding to the variation of surface potential, what can finally be measured as the variation of work function. These two specific effects are the base of the so-called Surface Photovoltage Spectroscopy (SPS) method [22] commonly used for many years to detect electronic states in the band gap originating from semiconductor surfaces commonly used for control of quality of semiconductor surfaces in aspect for their potential application in solid state microelectronics [23]. When using the photons of energy *hν* higher than band gap *E_bg_* (*hν* > *E_bg_*) this effect of variation of band bending (surface potential and subsequent work function) at the semiconductor surfaces is evidently stronger [22].

Fortunately, the SPV effect has been observed also at the surface of conductive oxides belonging to a group of oxide semiconductors, playing a role of the common gas sensor materials. It can be easily measured by using the commonly known Kelvin probe technique after external illumination of the gas sensor material playing the role of the measuring electrode.

It should be pointed out that, according to the available literature, recently there were only limited attempts of using the photovoltaic effects in gas detection. However, in these studies performed mainly by the group of Yamada [24,25,26], mistakenly called a surface photovoltage effect, only the photocurrent was measured across the MIS structures based on mesoporous silica films exposed to limited toxic gases like NO and NO_2_. Moreover, what is also puzzling, there has been only limited interest of using the true SPV effect even in the basic studies of gas interaction with MOX materials [27,28] without any reference to use this effect potentially for the toxic gas detection. This is why in our group, having significant experience in the use of very high sensitivity SPV effect for the control of quality of semiconductor surfaces in contact with different atmospheres [23] the studies focusing on the application of SPV effect for the control of interaction of toxic gases with selected MOX gas sensor materials have been lately carried out. As the result of these studies, a novel type of a room temperature gas sensor device utilizing a sensing mechanism based on the measurements of the SPV effect on porous nanostructures ZnO electrodes was developed.

In this paper we describe the proof-of-principle of the above mentioned SPV gas sensor system based on the Kelvin probe approach utilizing porous ZnO nanostructured thin films as the active electrode. As the first step, the fundamental physicochemical properties of the porous ZnO nanostructured thin films were analyzed with a special emphasis on the local surface chemistry and morphology, and were subsequently related to the interpretation of basic analytical sensing parameters of the elaborated novel type SPV gas sensor system.

## 2. Materials and Methods

As was mentioned above, in the constructed gas sensor device porous ZnO nanostructured thin films were used as the sensor material. They were fabricated using a two-step approach: first, nanostructured porous Zn films with a thickness of 3 μm were deposited onto Si (100) substrates by direct current (DC) reactive magnetron sputtering of a 99.95%-pure Zn target in an 6N argon/oxygen sputtering mixture at an oxygen concentration of 33% under total working pressures of 1.5 mTorr, for 2 h, using a Surrey NanoSystems reactor model 1000C (Surrey NanoSystems, Newhaven, UK). Subsequently, these porous Zn films were oxidized ex situ in a 6N-pure oxygen flow at 800 °C using a SHS-100 rapid thermal processing (RTP) furnace (Mattson Technology, Fremont, CA, USA) for 15 min in order to obtain the abovementioned nanostructured ZnO thin films. In such a form they were used as the above mentioned gas sensing electrode of an active area of 10 × 10 cm in our Kelvin type gas sensor system (device). Other technological details combined with the results of fundamental characterization of their bulk chemistry and morphology by chosen experimental methods performed at the Institute of Electron Technology, Warsaw, Poland, have been already described elsewhere [29,30].

Having in mind our recent experiences in studies of tin dioxide SnO_2_ thin films nanostructures as the commonly used oxide gas sensor material [31], the local surface chemistry of these porous ZnO nanostructured thin films was controlled by X-ray Photoelectron Spectroscopy (XPS) method at the Institute of Electronics, Silesian University of Technology, Gliwice, Poland. The experimental details of XPS studies have recently been described in our recent paper [32].

**Figure 1:**
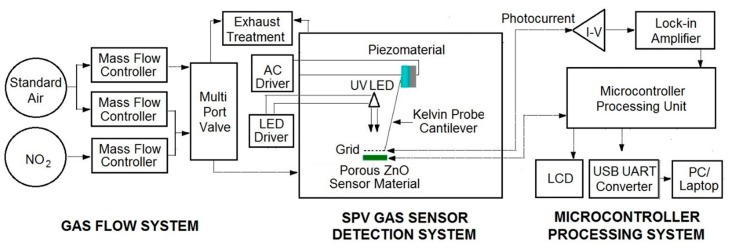
Simplified block-scheme of elaborated surface photovoltage (SPV) gas sensor system (device).

The elaborated surface photovoltage (SPV) gas sensor system consists of three main parts, as shown in Figure 1. Apart from the typical gas flow system containing selected gases, mass flow controllers with multiport valve, it contains the SPV gas sensor detection system combined with microcontroller processing system for data processing and acquisition. The elaborated SPV gas detection system is based on the reverse Kelvin probe flat type vibrating capacitor system. The capacitor subsystem consists of the porous ZnO nanostructured thin films as a flat gas sensor material playing the role of the active electrode, combined with the reference flat Cu metallic grid-type electrode on specific vibrating cantilever after piezoelectric driving using an AC voltage generator. 

The alternating SPV signal is measured as the variation of work function of the gas sensor material (with respect to the vibrating reference Cu electrode) as the result of its surface band bending variation after UV illumination by a UV5-400-30 type LED diode (Bivar Company, Irvine, CA, USA). It should be pointed out that the alternating SPV signal can also be measured using the static reference Cu electrode combined with UV LED light modulated by its respective driver. However, the latest approach does not allow the phenomena recognition that is responsible for the surface potential change. In other words, at the complex gas sensor system, the potential barrier is a sum of a fixed charge potential that may arise from the adsorbed species, strict surface region gas molecule reorganization, and carrier generation and redistribution in the SCR region.

For the measuring and acquisition of the SPV signal response in selected gases the microcontroller processing system is used containing the data processing control unit working with, among others, the I/V photocurrent converter (amplifier), the respective DAC and ADC converters, and finally the zero self-compensating lock-in amplifier for the reverse Kelvin probe flat vibrating capacitor system. Moreover, microcontroller processing system is equipped with USB to UART converter for laptop connection, enabling the use of our SPV gas sensor system as a mobile device. In our test experiments of the elaborated SPV gas sensor system (device) a mixture of pure nitrogen dioxide NO_2_ in standard synthetic air at different concentrations was used.

## 3. Results and Discussion

As was mentioned above in our SPV gas sensor system the porous ZnO nanostructured thin films deposited onto Si (100) substrate were used as the gas sensing material for which the bulk chemistry and morphology have been characterized by chosen experimental techniques [29] as shortly summarized below.

Using the Rutherford Backscattering Spectroscopy (RBS) it was shown that the obtained ZnO layers were almost stoichiometric with the oxygen to zinc atomic ratio close to 1. In turn, using the Scanning Electron Microscopy (SEM) it was observed from the cross-sectional images, that the obtained ZnO layers exhibit nanocoral morphologies as shown in [29]. Their polycrystalline character was also confirmed by X-ray diffraction (XRD) showing powder diffraction patterns, also described in [29]. However, since from our experience with SnO_2_ nanostructures we know that they display a significant surface non-stoichiometry additionally modified by the undesired strong surface carbon and water vapor contaminations, the additional XPS studies of the local surface chemistry our porous ZnO nanostructured thin films have additionally been performed. Such information is absolutely indispensable when trying to understand the gas sensing mechanism since the sensor effect appears just on the surface of the gas sensing materials at the depth related to Debye length [12,13], which is quite similar to the information depth of XPS method. The obtained XPS results are shortly described and interpreted below.

### 3.1. Local Surface Chemistry Of The Porous Zno Nanostructured Thin Films

**Figure 2:**
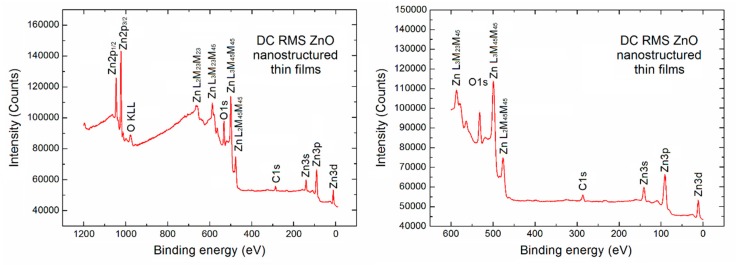
XPS survey spectra (full and in limited 600 eV BE range) of the ZnO nanostructured thin films used in our SPV gas sensor.

Initially, an XPS survey spectrum for the porous ZnO nanostructured thin films used in our SPV gas sensor device has been recorded, as shown in Figure 2 in two various binding energy ranges.

On the left side a typical full XPS survey spectrum in the binding energy (BE) range (1200 eV) is visible. It confirms that apart from the Auger electron lines (which can be usually skipped) the contribution from the basic elements of ZnO derived from the respected XPS core level Zn2p, O1s, Zn3s, Zn3p and Zn3d lines is observed. Moreover, what is crucial in our studies, an evident undesired contribution of the C1s XPS lines at BE ~286.0 eV is observed, what confirms that the strong undesired C contamination exists at the surface of porous ZnO nanostructured thin films used in our SPV gas sensor device.

Commonly, basing on the relative intensity (height) of Zn2p, O1s and C1s core level lines and the well-known analytical formula [33,34,35] and the respective atomic sensitivity factors related to the height of the above mentioned Zn2p, O1s and C1s peaks, the relative concentration of the selected elements can be calculated. However, because of the undesired high background in our observed XPS full survey spectrum of 1200 eV binding energy (BE) range with the additional useless Auger electron emission lines, the relative concentration of main elements at the surface of porous ZnO nanostructured thin films has also been estimated on the base of survey spectra in the limited binding energy range (600 eV) (right side in Figure 2).

Our calculation showed that our porous ZnO nanostructured thin films were rather far from the surface stoichiometry. The relative [O]/[Zn] and [C]/[Zn] concentration reached the values 0.63 and 0.31, respectively. It means that this is in an evident contrary to the information obtained by using the RBS method, where the oxygen to zinc atomic ratio was close to 1. This is probably related to the different information depth of both methods because for the RBS method it is at the level of hundreds of nm, whereas for XPS it corresponds only to the subsurface region of depth at the level of ~3 nm [33,34,35].

The different information depth was probably a reason that an evident C contamination was observed by XPS in our studies for our porous ZnO nanostructured thin films (with relative C]/[Zn] concentration of ~0.31), what was impossible by using the RBS method. This is why at the next step of our analysis we have focused on the local surface chemistry of our porous ZnO nanostructured thin films, with a special emphasis on the specific surface bondings. Our analysis was based on the deconvolution procedure of O1s and C1s spectral lines using the Casa XPS SPECS software. The obtained results are described below.

**Figure 3:**
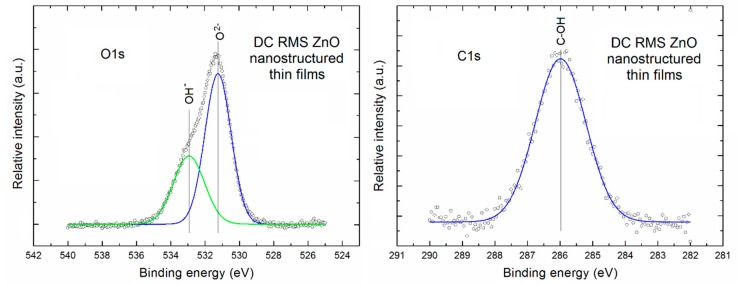
The XPS O1s and C1s lines after deconvolution using Gauss fitting procedure for the porous ZnO nanostructured thin films.

Figure 3 presents the XPS O1s and C1s lines after deconvolution using Gauss fitting (left and right column, respectively) for the porous ZnO nanostructured thin films.

It is clearly visible that the XPS O1s line of our porous ZnO nanostructured thin films is evidently asymmetric. After Gauss deconvolution (with very high line fitting (RMS ~0.998)) it consists of two evident components. A first one is located at the binding energy of ~531.2 eV and can be attributed to the O^2−^ ions in ZnO lattice of our porous ZnO nanostructured thin films. In turn, a second one located at binding energy ~532.9 eV can be attributed to the oxygen atoms in OH^−^ groups at the surface of our porous ZnO nanostructured thin films. The similar two above mentioned components of the O1s XPS line was recently observed, among other, by Gazia et [36] for the sponge-like nanostructured ZnO films deposited from the sputtered nanostructured zinc films. In addition, on the base of deconvoluted XPS O1s line the relative area of specific components in to O^2−^ ions with respect to the surface OH^−^ groups was determined as equal to 1.85. It directly means that even at the surface there is a domination of O^2−^ ions related to the ZnO crystalline lattice.

In turn, the XPS C1s line of our porous ZnO nanostructured thin films is practically symmetric. After its Gauss deconvolution (with high line fitting (RMS ~0.98)) it contains only one component always being observed at the binding energy of ~ 286 eV, what can be attributed to the C–OH surface bondings, commonly observed at the surface of various metal oxides, what is easy available in the NIST X-ray Photoelectron Spectroscopy Database [37].

Taking all above into account, which is crucial, it should be underlined that in our experiments the existence of undesired water vapor combined with carbon contaminations at the surface of our porous ZnO nanostructured thin films having the nanocrystalline, columnar structures of cross section in the range of several nm [29] has been confirmed. It cannot be ignored when their sensing properties are analyzed. This is crucial because this information, probably well undesired and commonly ignored in the available literature, will be additionally commented in the next chapter, where the gas sensor characteristics of our novel type gas sensor system (device) will be analyzed.

### 3.2. Gas Sensing Characteristics in NO_2_ Atmosphere

The gas detection effect is based on the variation of ZnO electrode surface charge and subsequent surface potential due to the interaction of toxic NO_2_ gas with our gas sensor material.

Already the primary experiments proved our expectations that our novel type SPV gas sensor system is working at room temperature. However, to reach the constant primary surface potential and subsequent constant starting value of the SPV signal allowing repeatable working conditions of our system, standard dry synthetic air was flowing through the system for several hours. Such pretreatment allowed for removing the adsorbed ambient water molecules from the electrode surface enabling a steady reference state for the sensing action. 

Subsequently, we have focused on the registration of the variation of SPV signal in response to changes of the concentration of the NO_2_ active gas in the standard dry synthetic air. During this proof-of-principle experiments, we have focused on two aspects, i.e., on determining the threshold gas concentration and sensor dynamic response.

**Figure 4:**
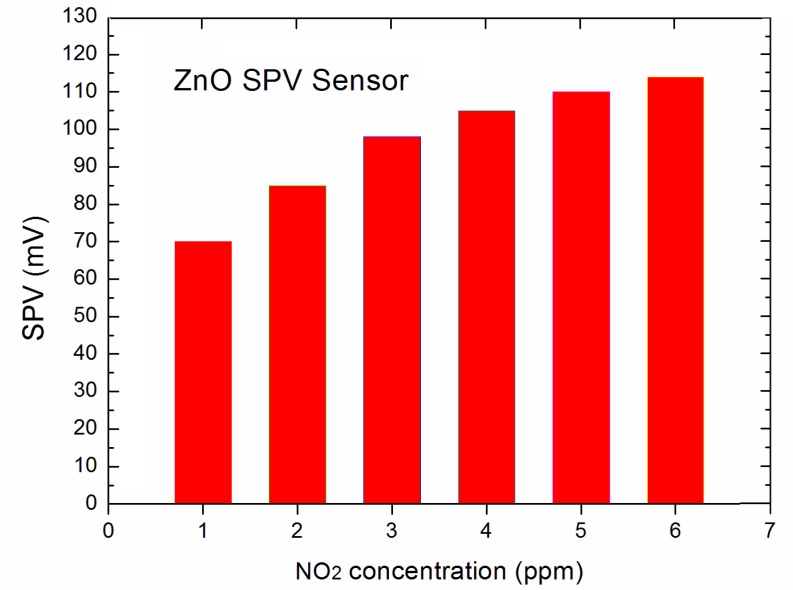
The variation of SPV signal as a function of NO_2_ concentration.

Our experiments showed that the elaborated novel type SPV gas sensor system allows the detection of NO_2_ gas up to 1 ppm, at the signal to noise (S/N) ratio at the level ~50. The obtained data for the lowest, selected NO_2_ gas concentration in the main range of 1 ÷ 5 ppm were presented in the form of a diagram shown in Figure 4.

At this point it should be underlined that perhaps our system is able to detect the NO_2_ gas detection below 1 ppm, since the signal to noise (S/N) ratio was at the level ~50, but our gas flow system (shown in Figure 1) was only able to work in repeatable and reliable measuring condition only up to 1 ppm. Nevertheless, the obtained results look very promising having in mind that our strong SPV signal even at 1 ppm of NO_2_ was reached already at room temperature working conditions. This performance is not achievable using the common resistive type gas sensor based on various forms of ZnO as gas sensor material [12,13]. Moreover, it should be also underlined that the respective, relative sensitivity is even at room temperature of about 1 order better with respect to the commonly used various forms of ZnO thin films working at higher temperatures, in the range of 200–350 °C [12,13]. It is an important advantage of our sensor prom the point of view of potential applications. As was mentioned above, at the next step of our experiments we have focused on the determination of dynamic characteristics of our SPV gas sensor system including response time and respected recovery time at specific NO_2_ concentrations.

**Figure 5:**
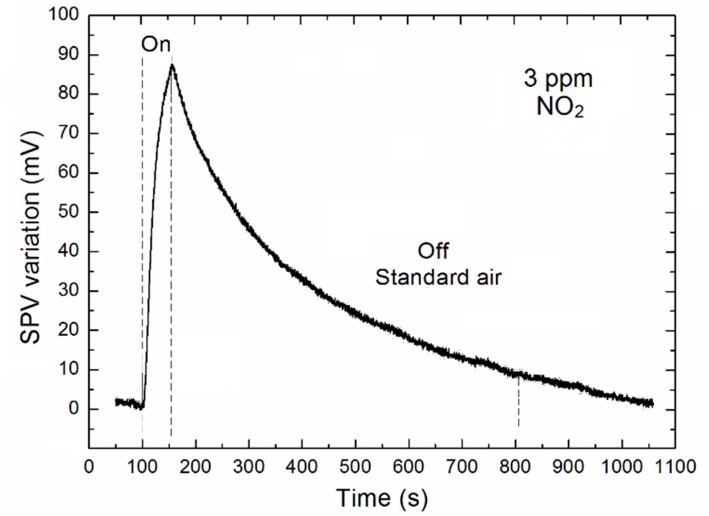
The variation of SPV signal as a function of time for the 3 ppm NO_2_ concentration.

Figure 5 shows the time dependent variation of the SPV signal related to the dynamic parameters for NO_2_ concentration of 3 ppm, in the middle of its main range used in our experiments. As it is commonly accepted in literature, the respective gas sensor dynamic characteristics (response and recovery times, respectively) are defined as the respective time at which the signal reaches 90% value of its full relative variation. Taking it into account it is clearly visible that for 3 ppm NO_2_ concentration the response time is at the level of about 50 s. Its estimated values for all other NO_2_ concentrations used in our experiments are summarized in Table 1. What is important, as mentioned above, the response time varies in the range of 52 ÷ 24 s for the variation of NO_2_ concentration the our main range of 1 ÷ 5 ppm, respectively. The second dynamic parameter, i.e., recovery time it is highly dependent on the concentration of the sensed gas and in the range of tens of minutes.

As mentioned above, the times related to the regeneration of our SPV gas sensor system still look rather long with respect to the commonly used the resistive type gas sensor based on various forms of ZnO as gas sensor material. However, it should be underlined that these values were already reached when only leaving the porous ZnO nanostructured thin films in a stream of synthetic air at room temperature, without any additional regeneration procedure. What is crucial, at this moment it can mainly be treated as the direct proof that the target gas (NO_2_) used in our experiments was only physically adsorbed onto the inner surfaces of the porous ZnO nanostructured thin films. In such a case a better dynamic characteristics could not be rather expected. Still, these responses are better than for the usual resistive sensors operating at room temperatures, at which they are usually close to the signal-to-noise ratio. Finally, they are promising for future coupling of the sensor with an additional electrode degassing mechanism (e.g., heat or light-based). The abovementioned regeneration effect can be additionally improved by using the additional IR-LED source. Our primary experiments are very promising in this aspect since the recovery time can be reduced by a factor of 5, however it would require further studies to optimize the degassing conditions.

## 4. Conclusions

In our studies a novel type room temperature surface photovoltage (SPV) gas sensor system based on the reverse Kelvin probe with vibrating grid type reference electrode and the porous ZnO nanostructured thin films as the active gas sensing electrode has been successfully developed.

The initial XPS experiments showed that for our porous nanostructured ZnO thin films an evident surface nonstoichiometry is observed combined with the high undesired H_2_O vapor and C surface contaminations. In turn, our gas sensing experiments showed, among others, that the elaborated surface photovoltage gas sensor system exhibits a relatively high sensitivity in NO_2_ atmosphere (up to 1 ppm at signal to noise ratio ~50) and relatively fast response time (~several seconds). Having identified undesired H_2_O vapor and C surface contaminations, we can see a potential for performance improvement, when the contaminants are removed. The presented device is significantly advantageous over the commonly used resistance type MOX gas sensor not only by using the nondestructive SPV effect measurements, but most importantly by operation at room temperature.

In currently ongoing work we aim at improving the sensor system performance, in particular by increasing the sensitivity and shortening the response and recovery times. We plan to test heat-cleaning of the electrode by additional IR-LED source and to study other ZnO/MOX nanostructures with extended internal surfaces for the more effective adsorption/desorption effects of target gasses during the gas sensor working conditions. 

## Figures and Tables

**Table 1:** Response time and respective recovery time of SPV signal of our SPV gas sensor device for the main range of NO_2_ concentration used in our experiments.

SPV Gas Sensor Dynamic Parameters	NO_2_ Concentration (ppm)				
	1	2	3	4	5
Response time (s)	52	42	38	30	24
Recovery time (s)	~1500	900	680	580	500
